# Numerical Simulation: Fluctuation in Background Synaptic Activity Regulates Synaptic Plasticity

**DOI:** 10.3389/fnsys.2021.771661

**Published:** 2021-11-22

**Authors:** Yuto Takeda, Katsuhiko Hata, Tokio Yamazaki, Masaki Kaneko, Osamu Yokoi, Chengta Tsai, Kazuo Umemura, Tetsuro Nikuni

**Affiliations:** ^1^Department of Physics, Tokyo University of Science, Tokyo, Japan; ^2^Department of Neuroscience, Research Center for Mathematical Medicine, Tokyo, Japan; ^3^Department of Sports and Medical Science, Kokushikan University, Tokyo, Japan; ^4^Graduate School of Emergency Medical System, Kokushikan University, Tokyo, Japan; ^5^KYB Medical Service Co., Ltd., Tokyo, Japan; ^6^The Institute of Physical Education, Kokushikan University, Tokyo, Japan

**Keywords:** frequency-dependent synaptic plasticity, background synaptic activity, fluctuation, calcium-based model, neural noise

## Abstract

Synaptic plasticity is vital for learning and memory in the brain. It consists of long-term potentiation (LTP) and long-term depression (LTD). Spike frequency is one of the major components of synaptic plasticity in the brain, a noisy environment. Recently, we mathematically analyzed the frequency-dependent synaptic plasticity (FDP) *in vivo* and found that LTP is more likely to occur with an increase in the frequency of background synaptic activity. Meanwhile, previous studies suggest statistical fluctuation in the amplitude of background synaptic activity. Little is understood, however, about its contribution to synaptic plasticity. To address this issue, we performed numerical simulations of a calcium-based synapse model. Then, we found attenuation of the tendency to become LTD due to an increase in the fluctuation of background synaptic activity, leading to an enhancement of synaptic weight. Our result suggests that the fluctuation affects synaptic plasticity in the brain.

## 1. Introduction

Synaptic plasticity is essential for information processing, including learning and memory. It consists of long term amplification and attenuation of the efficacy in synaptic transmission; long-term potentiation (LTP) and long-term depression (LTD) (Gerstner and Kistler, 2002). It has been thought that two mechanisms are involved in the synaptic plasticity. The first is a spike timing of the following presynapse and postsynapse. This spike-timing dependent plasticity (STDP) has been examined in numerous experimental and theoretical studies so far (Gerstner et al., 1996; Bi and Poo, 1998; Song et al., 2000). LTP is induced by presynaptic firing followed by postsynaptic firing occurring no more than tens of milliseconds, whereas postsynaptic firing preceding presynaptic spikes produces LTD (Markram et al., 1997; Bi and Poo, 1998; Debanne et al., 1998; Zhang et al., 1998; Feldman, 2000; Song et al., 2000). The second is a spike frequency (Bliss and Lomo, 1973; Bienenstock et al., 1982). High-frequency inputs in presynaptic neurons induce LTP, whereas the low-frequency firing induces LTD (Dudek and Bear, 1992; Mulkey and Malenka, 1992; Artola and Singer, 1993). This phenomenon is called frequency-dependent synaptic plasticity (FDP) and was formulated as a Bienenstock, Cooper, and Munro (BCM) rule (Bienenstock et al., 1982).

It has been reported that, in some cases, FDP may be more suitable than STDP for synaptic plasticity *in vivo*. The *in vivo* whole-cell patch-clamp recordings in rats revealed that a large amount of endogenous noise in the cerebral cortex limits strict spike timing, essential for STDP (London et al., 2010). A theoretical study also showed that changes in firing rates alone could sufficiently induce synaptic plasticity (Graupner et al., 2016). Moreover, some studies on STDP-based spiking neural networks considering dendritic and axonal propagation delays have suggested the importance of firing frequency (Madadi Asl et al., 2017, 2018).

Previously, we theoretically examined the FDP with *in vivo* conditions and demonstrated that the output of synaptic plasticity in neurons receiving even the same spike frequency varies by other multifaceted factors, that is, temporal spike patterns, calcium decay time constants and background activities (Hata et al., 2020). We notably showed that a rise in the frequency of background synaptic activity increases the probability of LTP, suggesting the importance of background synaptic activity on synaptic plasticity. Here, “background synaptic activity” is defined as factors other than EPSP (excitatory postsynaptic potential) that depolarize the postsynaptic membrane. This result qualitatively consists with preceding experimental studies (Stacey and Durand, 2001; Destexhe et al., 2003). Besides, it has been reported that neural noise including background synaptic activity contributes to information processing in the brain (Stein et al., 2005; Lucken et al., 2016; Uddin, 2020). However, the detailed involvement of the background synaptic activity in synaptic plasticity remains elusive. Remarkably, while the amplitude of the background synaptic activity usually fluctuates (Faisal et al., 2008), little is understood about the relation between the fluctuation in the background event and synaptic plasticity. In this study, we addressed this problem. We verified the association between the coefficient variation for the background noise amplitude and the synaptic plasticity by numerically analyzing a calcium-based model, one of the most suitable models for experimental results (Gerstner and Kistler, 2002; Shouval et al., 2002). We studied a synaptic model with calcium dynamics that mimics a thick-tufted layer 5 pyramidal neuron (TTL5 neuron) in the mammalian neocortex. The TTL5 neuron is considered one of the most extensively investigated neurons in the mammalian neocortex (Ramaswamy and Markram, 2015). It is suitable for understanding information processing processes and synaptic plasticity as a typical excitatory neuron. It has a pyramidal soma and a stereotyped dendritic morphology with protruding apical dendrites. Since the pyramidal cells in cortical layers 5–6, including TTL5 neurons, have been suggested to possess relatively long calcium decay time constants (80–100 ms), we fixed the calcium decay time constant at 80 ms for numerical calculations (Ahmed et al., 1998; Liu and Wang, 2001). The findings in our study may provide a better understanding of synaptic plasticity in neurons *in vivo* exposed to noisy background activity.

## 2. Methods

### 2.1. Model

We used a model for the FDP based on the calcium control hypothesis by Shouval et al. (2002). In this model, the postsynaptic calcium concentration affects the time derivative of the synaptic weight.

The dynamics of the synaptic weight *W*(*t*) is given as a function of the postsynaptic calcium concentration *Ca*(*t*) as follows:


(1) $$\begin{array}{l} \frac{d}{dt}W(t)=\eta (Ca(t))[\Omega (Ca(t))-W(t)]\;, \end{array}$$

where η(*Ca*(*t*)) and Ω(*Ca*(*t*)) are governed by


(2) $$\eta (Ca(t))={\left[\frac{p1}{p2+{\left(Ca(t)\right)}^{p3}}+p4\right]}^{-1}\;,$$

(3) $$\Omega (Ca(t))=1+4\text{sig}(Ca(t)-\alpha_{2},\beta_{2})-\text{sig}(Ca(t)-\alpha_{1},\beta_{1}),$$

(4) sig(x,β):  =exp(βx)/[1+exp(βx)].

The dynamics of the postsynaptic calcium concentration is described as follows:


(5) $$\frac{d}{dt}Ca(t)=I_{\text{NMDA}}(t)-\frac{1}{\tau_{ca}}Ca(t)\;,$$

(6) $$I_{\text{NMDA}}(t,V)=H(V)\left[I_{f}\Theta (t)e^{\left(-t/\tau_{f}\right)}+I_{s}\Theta (t)e^{\left(-t/\tau_{s}\right)}\right]\;,$$

(7) $$H(V)=-P_{0}\;G_{\text{NMDA}}\frac{\left(V-V_{r}\right)}{1+(Mg/3.57)\exp(-0.062V)}.$$

Here, τ_*ca*_ indicates the calcium decay time constant. In this study, we fixed τ_*Ca*_ = 80 ms, which is known as representative values in pyramidal cells in the deep cortex (layers 5–6) (Ahmed et al., 1998; Liu and Wang, 2001). The calcium current via the NMDA receptor (*I*_NMDA_) in Equations (5) and (6) is expressed as a function of time (*t*) and postsynaptic membrane potential (*V*). Θ(*t*) in Equation (6) is the Heaviside step function.

The postsynaptic membrane potential is given by


(8) $$V(t)=V_{\text{rest}}+V_{\text{epsp}}(t)+V_{\text{bg}}(t)\;,$$

(9) $$V_{\text{epsp}}(t)=\sum_{i}\Theta (t-t_{i})\left[e^{-(t-t_{i})/\tau_{1}}-e^{-(t-t_{i})/\tau_{2}}\right]\;,$$

where *V*_rest_ is the resting membrane potential and *V*_epsp_ is a depolarization term by EPSPs generated by binding glutamate to the postsynaptic AMPA receptors. *V*_bg_ is an applied membrane potential by the background synaptic activity, and described as follows:


(10) $$V_{\text{bg}}(t)=s\sum_{i}\Theta (t-t_{i})\xi_{\text{cv}}\left[e^{-(t-t_{i})/\tau_{1}}-e^{-(t-t_{i})/\tau_{2}}\right]\;,$$

where ξ_cv_ follows the normal distribution with average 〈ξ_cv_〉 = 1 and the coefficient of variation $CV=\langle {\left(\xi_{\text{cv}}-\langle \xi_{\text{cv}}\rangle \right)}^{2}\rangle /\langle \xi_{\text{cv}}\rangle$. The {*t*_*i*_} indicates Poisson processes with 1 Hz mean frequency generated for each simulation. We performed numerical simulations for three cases with different value of *CV* = 1, 3 and 5.

All model parameters in this study are listed below (Shouval et al., 2002):

*p*1 = 0.1 s, *p*2 = *p*1/10^−4^, *p*3 = 3, *p*4 = 1 s, $\alpha_{1}=0.35\;\mu \text{mol}/\text{d}{\text{m}}^{3}$, $\alpha_{2}=0.55\;\mu \text{mol}/\text{d}{\text{m}}^{3}$ and $\beta_{1}=\beta_{2}=80\;\mu \text{mol}/\text{d}{\text{m}}^{3}$, *I*_*f*_ = 0.75, *I*_*s*_ = 0.25, τ_*f*_ = 50 ms, and τ_*s*_ = 200 ms, *P*_0_ = 0.5, $G_{\text{NMDA}}=-1/140\;\mu \text{mol}\cdot {\text{dm}}^{\text{-3}}\text{/}(\text{m}\cdot \text{mV}),Mg=1$, *Mg* = 1, *Vr* = 130 mV, *V*_rest_ = −65 mV, *s* = 20 mV, τ_1_ = 50 ms and τ_2_ = 5 ms.

### 2.2. Numerical Simulations

In all numerical simulations, Wolfram Mathematica software version 12.0 was used. For each input frequency, we obtained the time evolution of the postsynaptic calcium concentration *Ca*(*t*) and the synaptic weight *W*(*t*) by numerically solving Equations (1)–(10). The inter-spike intervals for all inputs are constant.

**Figure 1:**
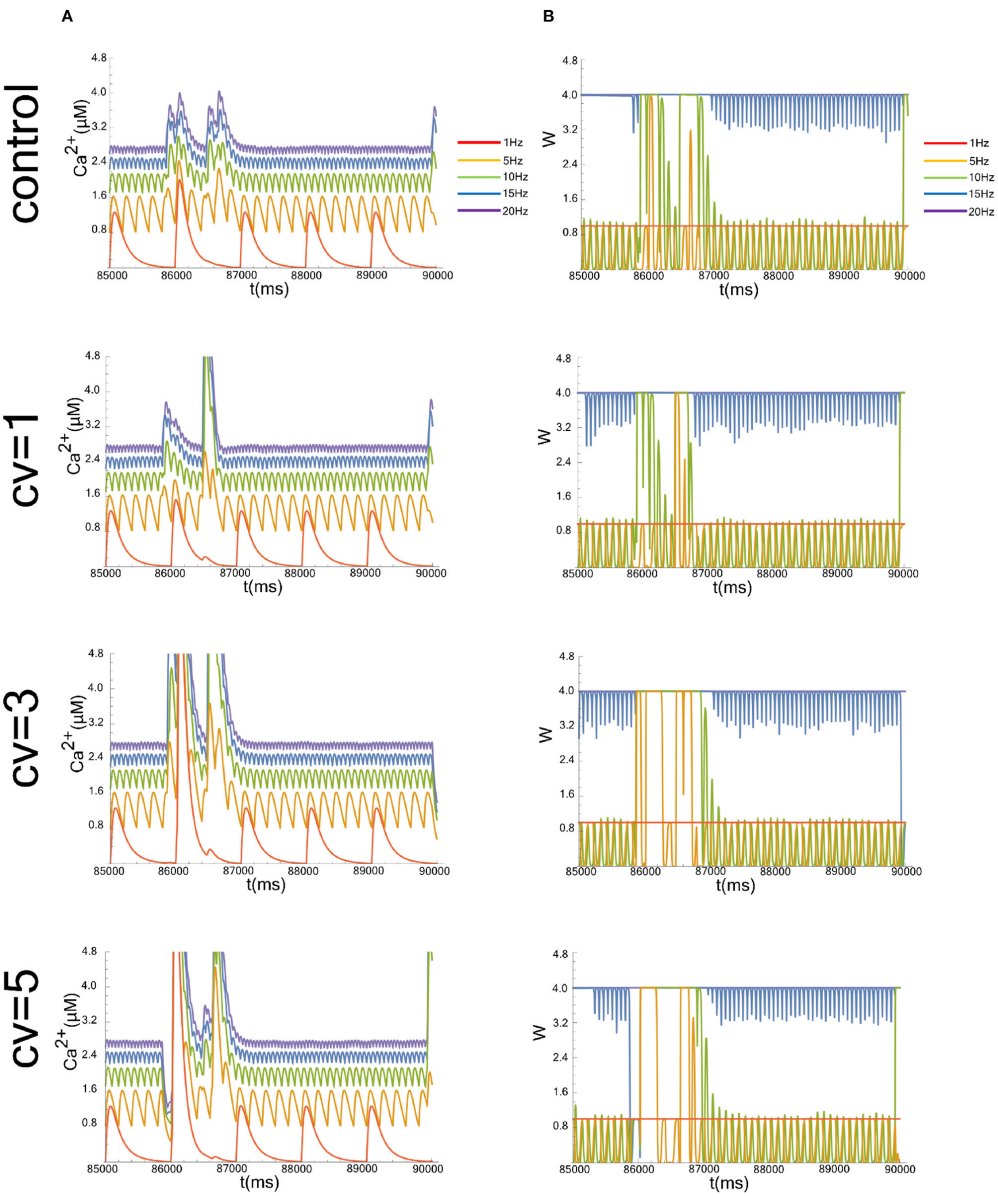
Typical simulation results, showing time evolution of the postsynaptic calcium concentration (*Ca*^2+^ in **A**) and synaptic weight (*W* in **B**). Figures start at 8.5 × 10^4^ ms to show a representative simulation result in the time interval over which the average is taken. We evaluated the case that the CV of the postsynaptic background activity (*V*_bg_), whose frequency is fixed at 1 Hz, were 1, 3, and 5. The data without the applied fluctuation is shown as a control. The graphs of the cases where the input frequencies are 1, 5, 10, 15, and 20 Hz are superimposed.

**Figure 2:**
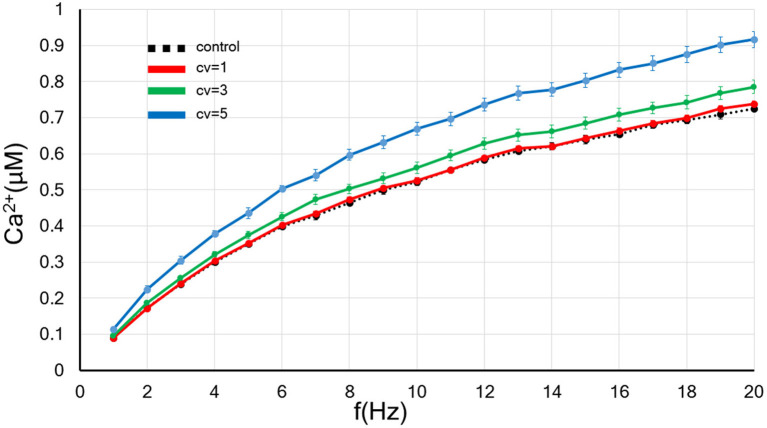
The CV of the amplitude for the background synaptic activity regulates the frequency-dependence of the postsynaptic calcium concentration. The *x*-axis shows the input frequency, and the *y*-axis indicates the postsynapitc calcium concentration. A black dotted line indicates the control without applied the fluctuation. The red, blue and green lines show the results of background synaptic activity with fluctuations in the *CV* = 1, 3, and 5. Error bars indicate the standard error of the mean (SEM).

**Figure 3:**
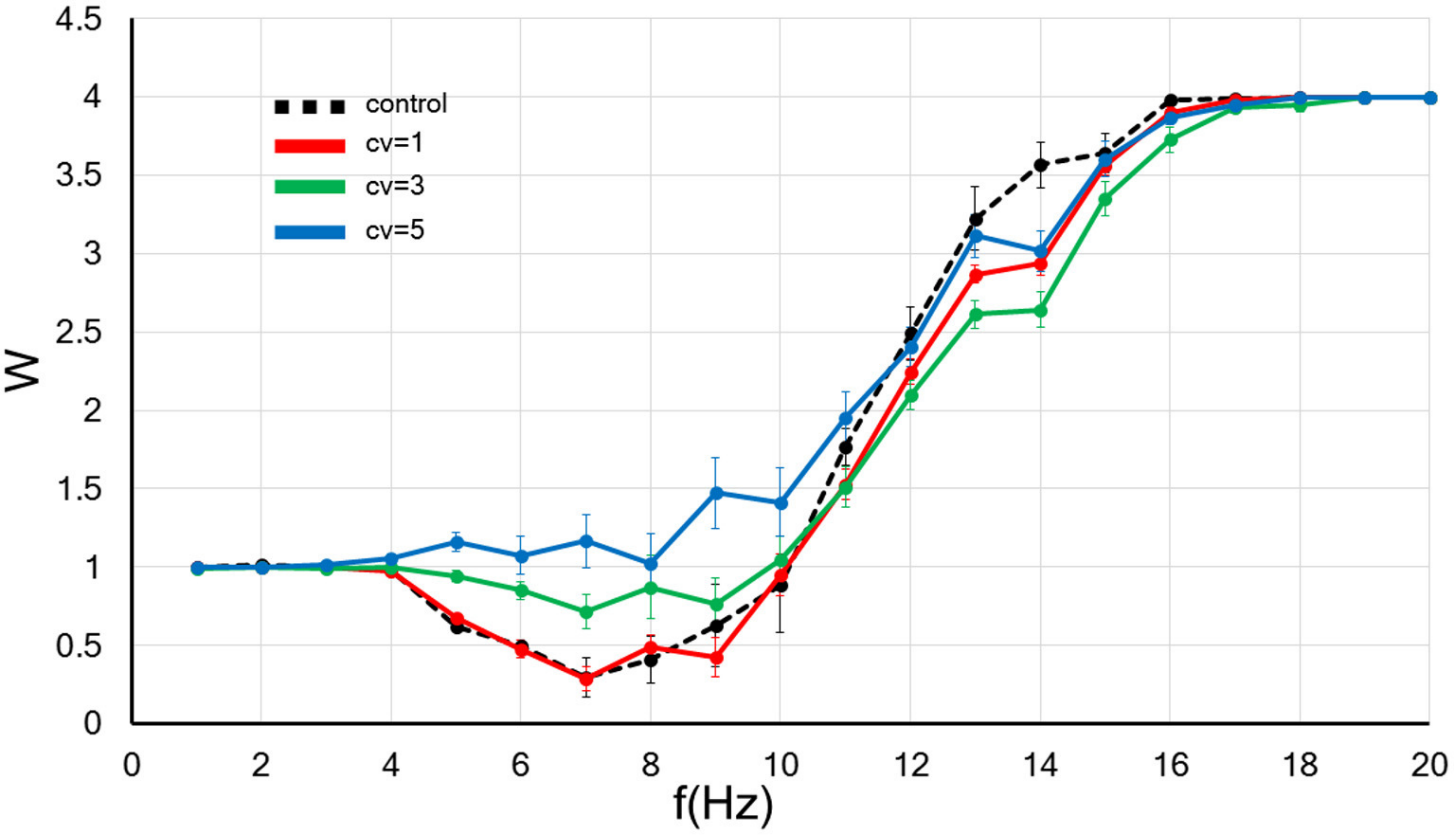
The CV of the amplitude for the background synaptic activity regulates the frequency-dependence of the synaptic strength. The *x*-axis shows the input frequency, and the *y*-axis indicates the relative synaptic weight. *W* < 1, *W* > 1, and *W* = 1 indicates the synaptic strength weakens, becomes more robust and do not change, respectively. A black dotted line indicates the control without applied the fluctuation. In contrast, the results of background synaptic activity with fluctuations in the *CV* = 1, 3, and 5 are shown by red, blue and green lines. Error bars indicate the standard error of the mean (SEM).

We gave fluctuations to the background synaptic activity as follows. First, we created five Poisson process time series with an average frequency of 1 Hz by changing the random seed (Hata et al., 2020). Next, to apply fluctuations per each CV (=1, 3 and 5) on the amplitude of each Poisson process stimuli, we generated Gaussian distributions consisting of three seeds for one CV for one Poisson process. The reason for creating three different Gaussian distributions for one CV is to average out the variability due to random species. The generated amplitude fluctuations were substituted into ξ_cv_ of Equation (10). Figure 1 and Supplementary Figure indicate representative simulation results in the interval over which the time average is taken. Figures 2, 3 show the plot of the means and standard deviations of these results of 15 experiments for each frequency.

To examine steady states of the system, we calculated the average of the calcium level or the synaptic weight between 8.5 × 10^4^ ms and 9.0 × 10^4^ ms. Data are expressed as the mean of ten independent experiments and the standard error of the mean (SEM).

## 3. Results

This research defines “background synaptic activity” as factors other than EPSP that depolarize the postsynaptic membrane. To approximate the calcium dynamics of the TTL5 neuron, we kept the calcium time constant at 80 ms for our numerical calculations. Previously, we demonstrated that the increase in the frequency of background synaptic activities induces the acceleration of the increased rate of the postsynaptic calcium level and the enhancement of synaptic weight (Hata et al., 2020). The present study aims to investigate the effect of fluctuation in background synaptic activity on the FDP.

We used a biophysical model by Shouval et al., in which the postsynaptic calcium concentration determines the change of the synaptic weight (Shouval et al., 2002; Shouval and Kalantzis, 2005). This model explains to a large extent experimental data of STDP and FDP obtained so far (Graupner and Brunel, 2018). An excitatory postsynaptic potential (EPSP) is generated when glutamate released from the presynapse binds to AMPA receptors, followed by depolarization of the postsynaptic membrane potential (*V*_epsp_ at Equations 8 and 9). The background synaptic activity also causes this depolarization of the membrane potential (*V*_bg_ at Equation 8). We varied the coefficient of variation of ξ_cv_ at Equation (10) to 1, 3, and 5 to examine the effect of varying the amplitude fluctuation range at a fixed frequency (1 Hz) on postsynaptic calcium concentration and synaptic plasticity. Calcium ions enter the neuron through channels regulated by NMDA receptors (NMDARs), only when the presynapse releases glutamate and the postsynaptic membrane is sufficiently depolarized (Equations 5–7). A moderate increase in postsynaptic calcium induces LTD, while the large increase leads to LTP (Equation 1). Thus, we applied presynaptic inputs ranging from 1 to 20 Hz and postsynaptic background activity fixed at 1 Hz to the synapse represented by this model.

We show representative time evolutions of the postsynaptic calcium concentration (*Ca*^2+^ in Figure 1A) and synaptic weight (*W* in Figure 1B). Simulations were performed for cases where the CV of *V*_bg_ were 1, 3, and 5, and where the input frequencies were 1, 5, 10, 15, and 20 Hz. Since we focused on the long-term behavior of *Ca*^2+^ and *W*, we calculated the average of the calcium level or the synaptic efficacy between 8.5 × 10^4^ ms and 9.0 × 10^4^ ms, which is sufficient for the system to reach a steady-state.

### 3.1. Relationship Between the Frequency-Dependence of Postsynaptic Calcium Concentration and the CV of an Amplitude for the Background Synaptic Activity

First, we examined the influence of the increase in the CV of amplitude for the background synaptic activity whose frequency was fixed at 1 Hz on the postsynaptic calcium level. As shown in Figure 2, the larger the CV, the greater the rate of increase in the postsynaptic calcium concentration. Thus, the rate up of the postsynaptic calcium concentration by increasing input frequency grew with an increase in the background synaptic activity fluctuation.

### 3.2. Relationship Between the Frequency-Dependency of Synaptic Weight and the CV of an Amplitude for the Background Synaptic Activity

Next, we examined the influence the increase in the CV of amplitude for the background synaptic activity on the synaptic weight. As before, we set the frequency of the background synaptic activity at 1 Hz. The numerical results are plotted in Figure 3.

**Figure 4:**
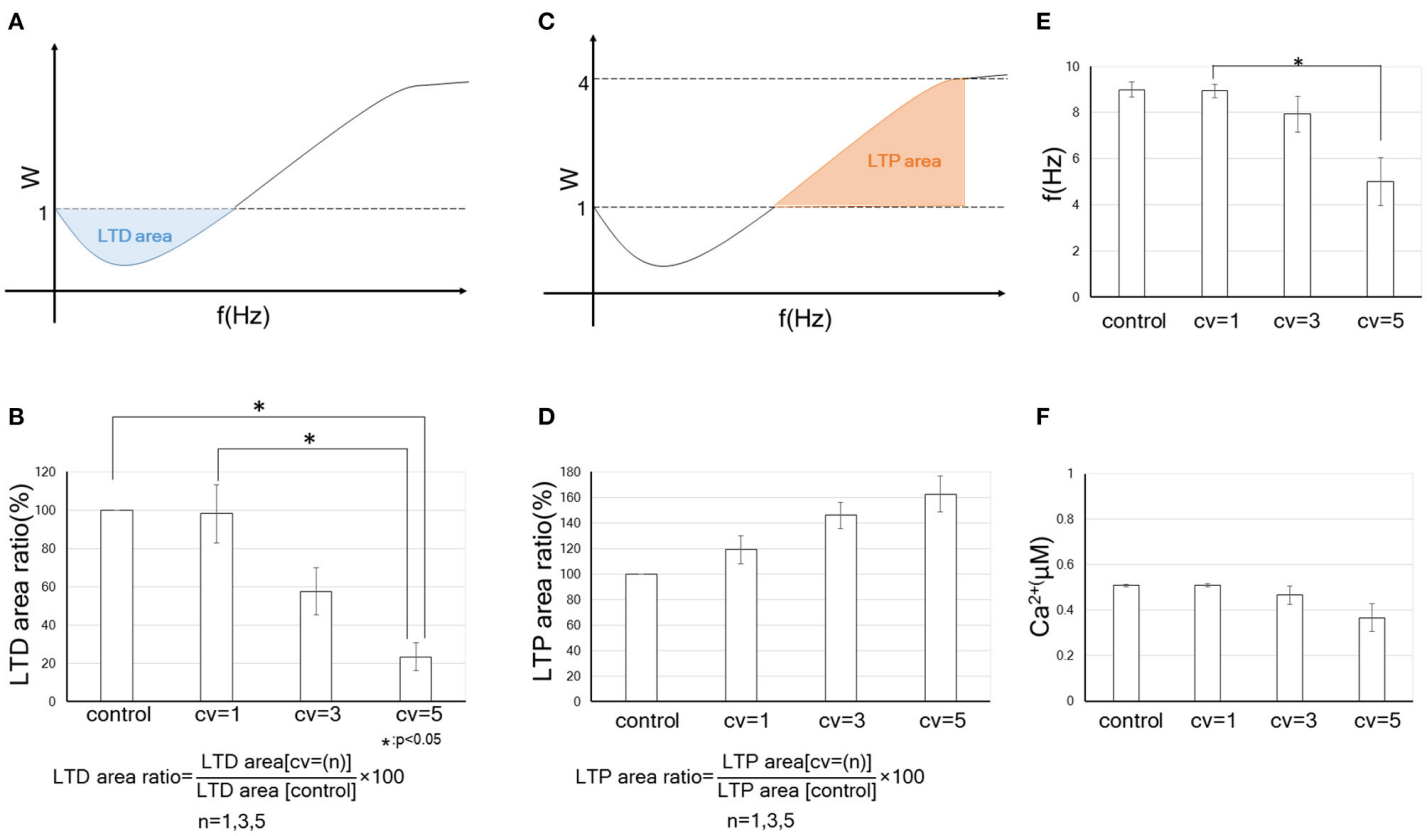
Quantitative data on the change in the synaptic weight by receiving fluctuated background synaptic activity. Error bars indicate the standard error of the mean (SEM). * indicates that the statistical test at the 5% level of significance is significant. **(A)** A schema of LTD-area. The blue part indicates the LTD-area, whose acreage is obtained by Equation (11). In **(B,D)**, control is without the fluctuation of background synaptic activity, while *CV* = 1, *CV* = 3 and *CV* = 5 each indicate the results of fluctuations in background synaptic activity with the corresponding CV. We defined “LTD-area ratio” or “LTP-area ratio” as the ratio of the LTD- or LTP- area with the fluctuation in the background synaptic activity and without the fluctuation (see formulas in **B,D**). The bar graph shows the LTD-area ratio (%) or LTP-area ratio (%) of the control and synapses receiving fluctuations of the corresponding CV. **(B)** Relation between the amplitude's fluctuation of background synaptic activity and tendency to become LTD. **(C)** A schema of LTP-area. The red part indicates the LTP-area, whose acreage is obtained by Equation (12). **(D)** Relation between the amplitude's fluctuation of background synaptic activity and tendency to become LTP. **(E)** LTD/LTP threshold (Hz) vs. CV of amplitude of background synaptic activity. **(F)** Relationship between postsynaptic calcium concentration at LTD/LTP threshold and amplitude CV of background synaptic activity.

To evaluate quantitatively the tendency to become LTP or LTD by changing the CV of the background synaptic activity, we defined “LTD-area” and “LTP-area.” As indicated at the schemas in Figures 4A,C, they are given as:


(11) $$LTD\;area:\;=\int_{0}^{f_{0}}W(f)df,\;$$

(12) $$LTP\;area:\;=\int_{f_{0}}^{f_{+}}W(f)df.\;$$

Here, *f*_0_ in Equations (11) and (12) indicates the LTD/LTP threshold, which is defined as the frequency at which the synaptic strength first returns to 1 after falling below 1 when the input frequency increases from 0 Hz. In Equation (12), *f*_+_ is defined as the smaller value of 20 Hz and the frequency reaching a plateau. Furthermore, to quantify the effect of the fluctuation in the amplitude for the background synaptic activity on synaptic plasticity comparing the control, we calculated “LTD-area ratio” and “LTP-area ratio” (which are obtained by equations in Figures 4B,D). We found that the LTD-area ratio decreased as the fluctuation increased. In particular, when *CV* = 5, the LTD-area ratio became significantly smaller than when control and *CV* = 1. On the other hand, the LTP-area ratio tended to go up as the CV increased, but it was not significant. Thus, an increase in the fluctuation of the background synaptic activity leads to the enhancement of synaptic efficacy mainly through decreasing in the LTD phase. These results suggest that the FDP output (LTP or LTD) varies depending on the size of the fluctuation for background synaptic activity, even if the mean amplitudes and frequency of it is the same.

We examined the association of LTD/LTP thresholds with fluctuations in background synaptic activity. As illustrated in Figure 4E, LTD/LTP thresholds become significantly smaller with the increase in the fluctuation of background synaptic activity. The postsynaptic calcium concentration at the LTD/LTP threshold also tends to be smaller with the CV and not to be constant (Figure 4F). These results suggest that some variable besides calcium concentration may be involved in the destiny (depression or potentiation) of synaptic plasticity. It could be a dynamic factor such as the time derivative of calcium concentration. Using cerebellar parallel fiber-Purkinje cell synapses, Piochon et al. found that not the intracellular calcium concentration but its temporal variations determine the output of synaptic plasticity. This mechanism involves autophosphorylation of CaMKII, which is the most well-studied effector in synaptic plasticity (Piochon et al., 2016). Although the BCM curve of cerebral cortical neurons is different from that of cerebellar cells in having a reversed sign shape, the studies by us and Piochon et al. are similar in suggesting that the calcium dynamics, rather than its concentration *per se*, determine the fate of synaptic plasticity. It is interesting from a metaplasticity point of view that our results suggest qualitatively similar phenomena to the Piochon et al.'s study, even though the model in this study does not explicitly include CaMKII. Analysis of the model, including the effects of CaMKII, may further clarify the relevance of background synaptic activity to calcium dynamics and synaptic plasticity (Graupner and Brunel, 2007).

## 4. Discussion

The postsynapse *in vivo* receives intense background synaptic activity, which is defined as factors except for EPSPs that induce depolarization and fluctuations in the membrane potential. Background synaptic activity is considered to be composed of at least four components: spontaneous miniature postsynaptic currents (MPSCs), (back-propagating) dendritic action potentials (BPAPs and DAPs), stochastic properties of ion channels and thermodynamical phenomenon in the cell membrane (Aizawa et al., 1975; White et al., 2000; Destexhe et al., 2003; Faisal et al., 2008; Kavalali et al., 2011; Poznanski and Cacha, 2012). MPSCs are produced by the spontaneous release of neurotransmitters and observed even in the absence of presynaptic input (Fatt and Katz, 1950). BPAPs are the action potentials that back-propagate into the dendritic tree. Spikes also arise within local dendrites (DAPs). The random opening and closing of ion channels in resting-state results in electrical currents, inducing membrane potential fluctuations without presynaptic inputs (White et al., 2000). Although little is known about the thermodynamic properties of neurons, it has been suggested that the action potential forces the membrane through the transition from fluid to gel state, leading to the membrane potential change (Aizawa et al., 1975; Heimburg and Jackson, 2005; Andersen et al., 2009; Fillafer et al., 2021).

At a glance, such background activity seems not to play a significant role in synaptic plasticity. Still, several experimental and theoretical studies have demonstrated that an appropriate level of background synaptic activity enhances the ability for neural signal detection and regulates neural plasticity (Stacey and Durand, 2001; Destexhe et al., 2003; Lu et al., 2019). Many studies have revealed that MPSCs regulate postsynaptic responsiveness in homeostatic synaptic plasticity (Sutton et al., 2006; Lee et al., 2010; Kavalali et al., 2011). BPAP is also important for synaptic plasticity (Destexhe et al., 2003; Remy and Spruston, 2007). Even a single presynaptic burst induces LTP with appropriate BPAPs in hippocampal synapses (Remy and Spruston, 2007). Some neurological disorders linked to neural plasticity are thought to be caused by background synaptic noise (Davis and Plaisted-Grant, 2015; Hancock et al., 2017). Further, our previous study indicated that an increase in the frequency of the background synaptic activity is likely to cause LTP (Hata et al., 2020). Thus, background synaptic activity might be beneficial to neural information processing. However, the detailed association between the background synaptic activity and neural plasticity remains unclear. Here, we focused on fluctuation in the amplitude of background synaptic activity. We found that it enhances synaptic connections even if the frequency in both presynaptic input and the background activity does not change, as you can see Figure 4B showing an increase in fluctuation width of the background activity significantly attenuates the LTD phase.

So what factors influence fluctuations in background synaptic activity? First, statistical properties of vesicular release can be considered one factor in the CV of fluctuations in background synaptic activity. Several experimental research found synaptic vesicular release counts follow a binomial distribution (Pulido et al., 2015; Malagon et al., 2016). The maximum number of vesicles per synapse that affects the CV of fluctuation in postsynaptic membrane potential is constrained by the number of presynaptic docking sites (Miki et al., 2017). The number of the docking site is in proportion to the number of clusters of voltage-gated calcium channels in the active zone. Both parameters depend on the developmental stage of animals and synaptic size (Nakamura et al., 2015; Miki et al., 2017). Further, the clusters of the calcium channels were individually detected in many kinds of synapses.

Next, dendritic action potentials (DAPs) can also be one source of CVs of fluctuations in background synaptic activity. In a study of intracellular recordings from the distal dendrites in pyramidal cells of free-behaving rats, the spike rate of DAP and subthreshold dendritic membrane fluctuations were several-fold larger than those of the cell body, suggesting that dendritic activity, including postsynaptic activity, has a significant role in neural computation (Moore et al., 2017). It is more prominent in exploratory behavior than in sleep, implying that the dendritic spike rate and fluctuation in postsynaptic membrane potential increase with elevated central nervous system (CNS) activity. Taking into account this result (Moore et al., 2017), our previous study (Hata et al., 2020) and current findings (Figures 3, 4), it is suggested that elevation of the CNS activity upregulates synaptic efficacy through an increase in the frequency and fluctuation in the amplitude of background synaptic activity. Moreover, Ordemann et al. showed that dendritic spike is less likely to occur, and thereby LTP is less likely to be induced, in fmr1 KO mice, an animal model of fragile X syndrome (FXS), which exhibits cognitive dysfunction and autism spectrum disorder (ASD) (Ordemann et al., 2021). This study supports our results by suggesting that appropriate background synaptic activity is necessary for LTP induction. It would be interesting to explore if background synaptic activity is associated with the pathogenesis of cognitive impairment and ASD. In examining this issue, it may be helpful to divide the background synaptic activity into the frequency and the amplitude fluctuations. The reason is, our theoretical analysis indicates that an increase in the frequency of background synaptic activity facilitates LTP (Hata et al., 2020), whereas an increase in fluctuation in postsynaptic membranes primarily increases synaptic weight by making LTD less likely to occur. The molecular mechanisms underlying these differences and their implications for synaptic plasticity will require further study.

Thus, the CV of fluctuations in background synaptic activity differs depending on anatomical and physiological features, suggesting that fluctuations in the amplitude of background synaptic activity are involved in the plasticity properties in individual neurons (Holderith et al., 2012; Nakamura et al., 2015).

As shown in Equation (5), we only consider calcium influx via NMDARs in this study. Certainly, synaptic plasticity in the mammalian CNS is thought to depend on the activation of NMDARs mainly. Still, several non-NMDAR-dependent forms of synaptic plasticity have also been identified in various areas of the CNS (Alkadhi, 2021). NMDAR-independent synaptic plasticity can be classified by calcium influx pathways: calcium-permeable glutamate AMPA receptor (CP-AMPAR), L-type voltage-gated calcium channel (L-type VDCC), and Ca2+ release from intracellular stores. GluA2 subunit-deficient CP-AMPAR is a pivotal regulator of LTD and LTP. CP-AMPARs are rarely present at synapses in normal conditions. Under physiologically and pathophysiologically specific situations, however, they are recruited to synapses and play an important role in modifying synaptic signaling (Liu and Zukin, 2007; Whitehead et al., 2013; Purkey and Dell'Acqua, 2020). Interestingly, presynaptic input and hyperpolarization of the postsynaptic membrane cause CP-AMPAR-dependent LTP, which is blocked by the depolarization of it Lamsa et al. (2007), Alkadhi (2021). Thus, by considering a functional form such that the slope of Equation (7) is negative and analyzing similarly to the present one, we may find out how CP-AMPAR-induced anti-Hevian synaptic plasticity is involved in CNS disorders like acute stress, ischemia, spinal cord injury, and neurodegenerative diseases.

L-type VDCCs are also significant players in NMDAR-independent plasticity. L-type VDCC-dependent form of LTP is induced in the glutamatergic synapses of CA1 and CA3 in the hippocampus, lateral amygdala and dorsal raphe nucleus (Alkadhi, 2021). In the lateral amygdala, pairing weak presynaptic stimulation with strong postsynaptic depolarization induces LTP depending on L-type VDCCs not on NMDARs (Bauer et al., 2002). In contrast, 30 Hz tetanus causes an NMDAR-dependent, L-type VDCC-independent form of LTP. This phenomenon may be understood by taking into account the I-V relatlon of NMDAR and L-type VDCCs. The reverse potential of NMDAR is 0 mV, while the inward currents of L-type VDCC are maintained at 0 mV (Spruston et al., 1995; Thibault and Landfield, 1996). It suggests that during strong depolarization of the postsynaptic membrane, L-type VDCCs are more likely to be the source of the postsynaptic intracellular calcium than NMDARs. Considering them with our results, L-type VDCCs may be dominant when the postsynapse depolarizes by the background synaptic activity frequency increases, and NMDARs may be prevalent when the postsynaptic membrane potential fluctuations are large but the depolarization does not occur. There are many unexplained issues regarding the switching between NMDARs and L-type VDCCs as calcium source. Further research is needed to clarify this point.

The regulator of intracellular calcium stores for synaptic plasticity has been identified as ryanodine receptors (RyRs) and inositol (1,4,5)-trisphosphate receptors (IP3Rs) (Baker et al., 2013). These factors cooperating with membrane calcium channels, NMDARs and L-type VDCCs are essential in promoting biochemical pathways associated with synaptic plasticity. The biochemical responses by the background synaptic activity are largely unexplained and need to be further elucidated.

Our findings may be relevant to the physiology and pathophysiology of the brain, which is regarded as a neural network. Theories integrating sleep and synaptic homeostasis argue that synaptic potentiation occurs more readily during wakefulness, while slow-wave oscillation (0.5–4.5 Hz) during sleep leads to LTD (Tononi and Cirelli, 2003, 2014). Our findings strengthen this argument by considering that during wakefulness, the level of background synaptic activity rises as the activity level of many regions of the brain increases compared to that during sleep. Moreover, since homeostatic plasticity usually results in a homeostatic level of firing throughout the brain, it also makes sense that the degree of fluctuation, rather than the frequency of background synaptic activity, regulates whether LTP or LTD occurs.

Psychological experiments and animal model studies with ASD patients indicate patients' different sensitivity patterns to sensory stimuli than healthy controls. This difference is suggested to be associated with reduced neural noise, and an appropriate amount of endogenous noise is proposed to be required to integrate multiple sensory information in the brain (Greenaway et al., 2013; Davis and Plaisted-Grant, 2015). The present results may provide further insight into the pathogenesis of ASD in the context of metaplasticity. The current results could help to clarify the pathogenesis of ASD in the context of metaplasticity. We have also previously shown by the neural network model that peri-injury activity enhances after brain injury. The variance of membrane potential is more significant following injury than before (Kitano et al., 2015). Similar findings have been validated experimentally, where neuroplasticity after brain injury results in the remapping of sensory and motor representations in the cortex (Guerriero et al., 2015). Since we only examined excitatory fluctuations in the present study, by incorporating inhibitory elements, it will be possible to explore synaptic plasticity after injury mathematically. Progress in these researches may lead to the establishment of methods for recovering from central nervous system injuries that are considered challenging (Hata et al., 2006a,b).

We discuss the limitations and prospects of this study below.

(1) This study is a theoretical study without experiments. By numerically calculating the calcium-based model with fluctuations applied to the background synaptic activity, we obtained novel conclusions consistent with previous studies. However, they are just theoretical predictions and should be verified in future experiments.
(2) The background synaptic activity components, the DAP and BPAP, have been identified as only an excitatory type. The other elements (MPSCs, stochastic channel opening and thermal noise) possibly hyperpolarize the membrane potential. For example, there are inhibitory as well as the excitatory type of MPSCs (Ropert et al., 1990; Gao et al., 2017). In this study, we focused only on the excitatory background activity according to our previous study (Hata et al., 2020). However, the relationship between inhibitory background synaptic activity and synaptic plasticity remains to be elucidated. It would be meaningful to study this issue.
(3) Since we focused on synaptic plasticity on a long time scale (more than tens of minutes), we did not consider short-term plasticity (sub-second scale). Presynaptic events are involved in short-term plasticity (STP), which includes short-term depression (STD) and short-term facilitation (STF) (Markram and Tsodyks, 1996; Abbott et al., 1997). On the other hand, the calcium-based model we used in this study only accounts for postsynaptic phenomena. STP is a process in which postsynapses respond depending on the presynaptic activity history. It is crucial for sensorimotor processing, learning and cognition on sub-second time scales (Motanis et al., 2018). STD occurs by the depletion of neurotransmitters expended in the synaptic transmission chain, while the mechanism of STF involves an increase in the probability of neurotransmitter release triggered by the upregulation of presynaptic calcium signaling (Zucker and Regehr, 2002; Jackman et al., 2016; Motanis et al., 2018). Thus, presynaptic events are essential for STP. Furthermore, recent studies have proposed that STP influences long-term synaptic plasticity. A theoretical analysis showed that integrating STP into the long-term plasticity improves a signal-to-noise ratio to the neural stimulus (Costa et al., 2015). In the future, the role of background synaptic activity in STP-incorporated long-term plasticity should be elucidated.

In conclusion, we examined the effect of fluctuations in background synaptic activity on synaptic plasticity by numerically solving a calcium-based model. We found that the LTD phase in FDP decreases significantly with an increase in the fluctuation of the background activity, leading to the strengthening of the synaptic efficacy. Thus, we newly found that the amplitude fluctuation of background synaptic activity has a positive effect on the output of FDP.

## Data Availability Statement

The original contributions presented in the study are included in the article/Supplementary Material, further inquiries can be directed to the corresponding author.

## Author Contributions

KH conceived the presented idea and developed the theory. YT, KH, and TY designed the model and the computational framework. YT, TY, and OY performed the numerical calculation. YT, KH, TY, MK, CT, KU, and TN analyzed the data. KH and TN prepared the manuscript draft. All authors reviewed the manuscript.

## Funding

This work was supported by JSPS KAKENHI Grant Numbers JP18K10858, 21H03327.

## Conflict of Interest

MK was employed by KYB Medical Service Co., Ltd. The remaining authors declare that the research was conducted in the absence of any commercial or financial relationships that could be construed as a potential conflict of interest.

## Publisher's Note

All claims expressed in this article are solely those of the authors and do not necessarily represent those of their affiliated organizations, or those of the publisher, the editors and the reviewers. Any product that may be evaluated in this article, or claim that may be made by its manufacturer, is not guaranteed or endorsed by the publisher.
