# Supplementary material for: Numerical Simulation: Fluctuation in Background Synaptic Activity Regulates Synaptic Plasticity
Source: Front Syst Neurosci. 2021 Nov 22;15:771661. doi: 10.3389/fnsys.2021.771661 (PMC8646040; doi:10.3389/fnsys.2021.771661)
Supplement: Supplementary file 1 [file Image_1.pdf]

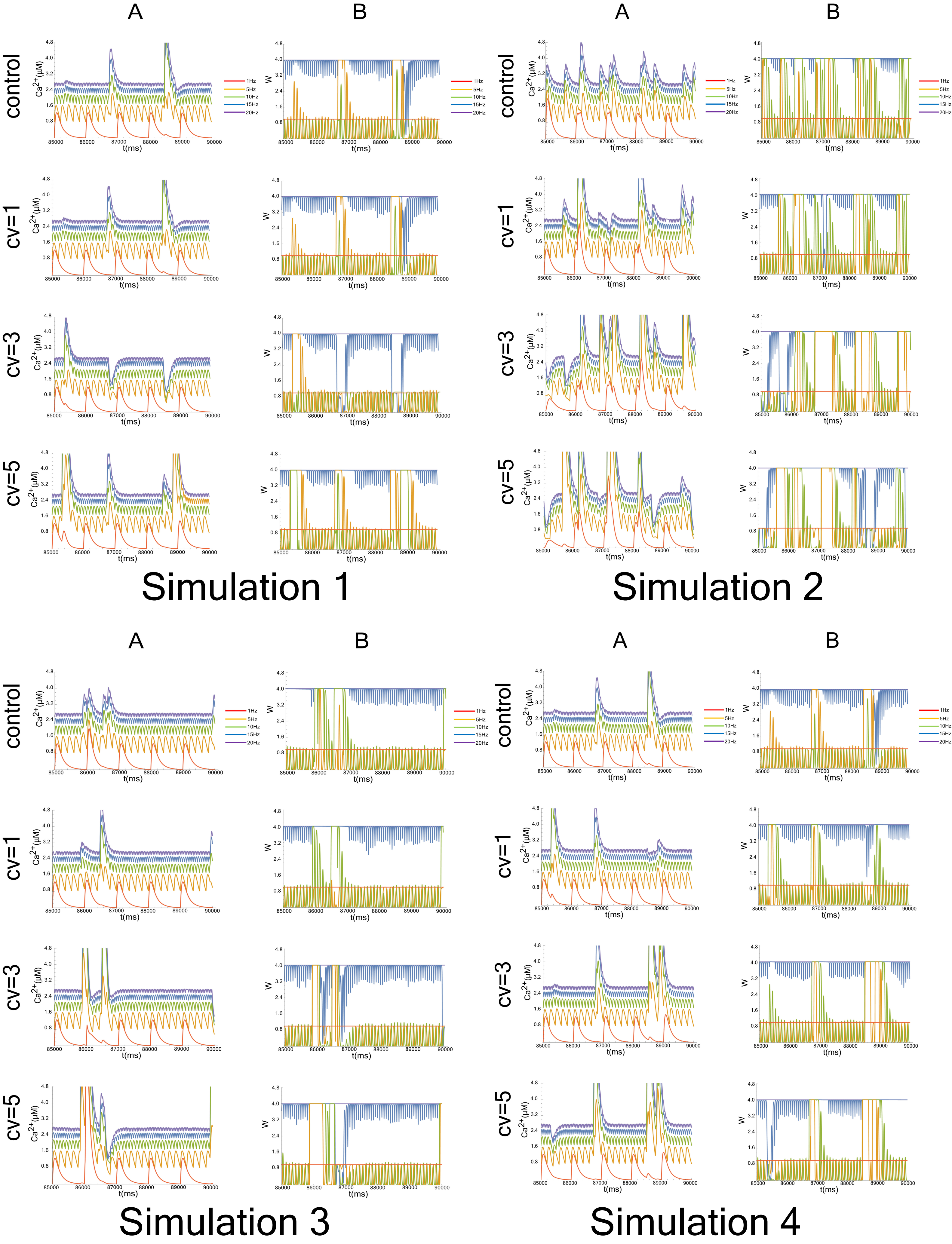

Supplementary Figure

Representative simulation results in the interval over which the time average is taken.
